# Supplementary material for: A randomized trial to evaluate a complex, co-created, culture-sensitive intervention to promote healthy lifestyles and compliance to therapy in immigrants with type 2 diabetes: A protocol of a multicenter Italian study
Source: PLoS One. 2025 Feb 24;20(2):e0317994. doi: 10.1371/journal.pone.0317994 (PMC11849826; doi:10.1371/journal.pone.0317994)
Supplement: S1 Table — (PDF) [file pone.0317994.s001.pdf]

Table 1 Elements of the intervention by topic and flexibility (degree of freedom of co-creation approach) in the definition

TOPIC	MANDATORY ELEMENTS	OPTIONAL ELEMENTS	CO-CREATION MODULAR ELEMENTS
**Diet**	Individual interviews with dietitian	- Number and scheduling of individual interviews, involved operators<br>- Setting and access mode to the interviews	- Aspects and topics to investigate and discuss with analysis tool in order to tailor the intervention<br>- Use of portion sizes methods<br>- Use of intercultural calendar to simplify lifestyle adjustments at particular times of the year<br>- Get in touch in preparation for the interview, including possible indication to produce supporting image material
	Group sessions about nutrition	- Number and scheduling of group sessions about each topic, involved operators, beneficiaries (e.g. patients, relatives, caregivers)<br>- Setting and access mode to the sessions	- Adjustment of the planning and the methods of operation of group sessions<br>- Figures to involve<br>- Aspects and topics to investigate and discuss
	Use of language materials	- Ways of presenting and using the materials	- Selection of most appropriate materials
**Physical activity**	Work groups about physical activity	- Number and scheduling of group sessions about each topic, involved operators, beneficiaries (e.g. patients, relatives, caregivers)<br>- Contents<br>- Setting and access mode to the sessions	- Adjustment of the planning and the methods of operation of group sessions<br>- Figures to involve<br>- Aspects and topics to investigate and discuss<br>- Use of potential local resources as map of opportunities
	Use of language materials	- Ways of presenting and using the materials	- Selection of most appropriate materials
**Therapeutic adherence**	Focus on the connection between pharmacological therapy and lifestyle during individual interviews	- Ways of developing this topic during individual interviews and group sessions	- Critical issues to include in the discussione about this topic
	Use of language materials	- Ways of presenting and using the materials	- Selection of most appropriate materials
**TOPIC CROSS-CUTTING ACTIVITIES**			
**LINGUISTIC and CULTURAL MEDIATION**	Cultural mediation activation	- Cultural mediation activation mode<br>- Number of mediators to involve	- Type of mediators to involve<br>- Setting/methods of delivery of mediation<br>- Modes of interaction between operators and mediators
**EDUCATION**	Operators and mediators training on effective communication and comprehension assessment	- Number of operators and mediators to involve (ideally those more representative in the intervention additional component)	- Education contents<br>- Most critical issues to cover during education activities
	Cultural mediators education about diabetes management	- Number of mediators to involve (ideally those more representative in the intervention additional component)	- Most critical issues to cover during education activities
	Peer to peer education and/or education with operators on cultural aspects nutritionally connected	- Ways of presentig education activities, users and operators to involve	- Selection of topics and contents
