# Supplementary figures and images for: A randomized trial to evaluate a complex, co-created, culture-sensitive intervention to promote healthy lifestyles and compliance to therapy in immigrants with type 2 diabetes: A protocol of a multicenter Italian study
Source: PLoS One. 2025 Feb 24;20(2):e0317994. doi: 10.1371/journal.pone.0317994 (PMC11849826; doi:10.1371/journal.pone.0317994)

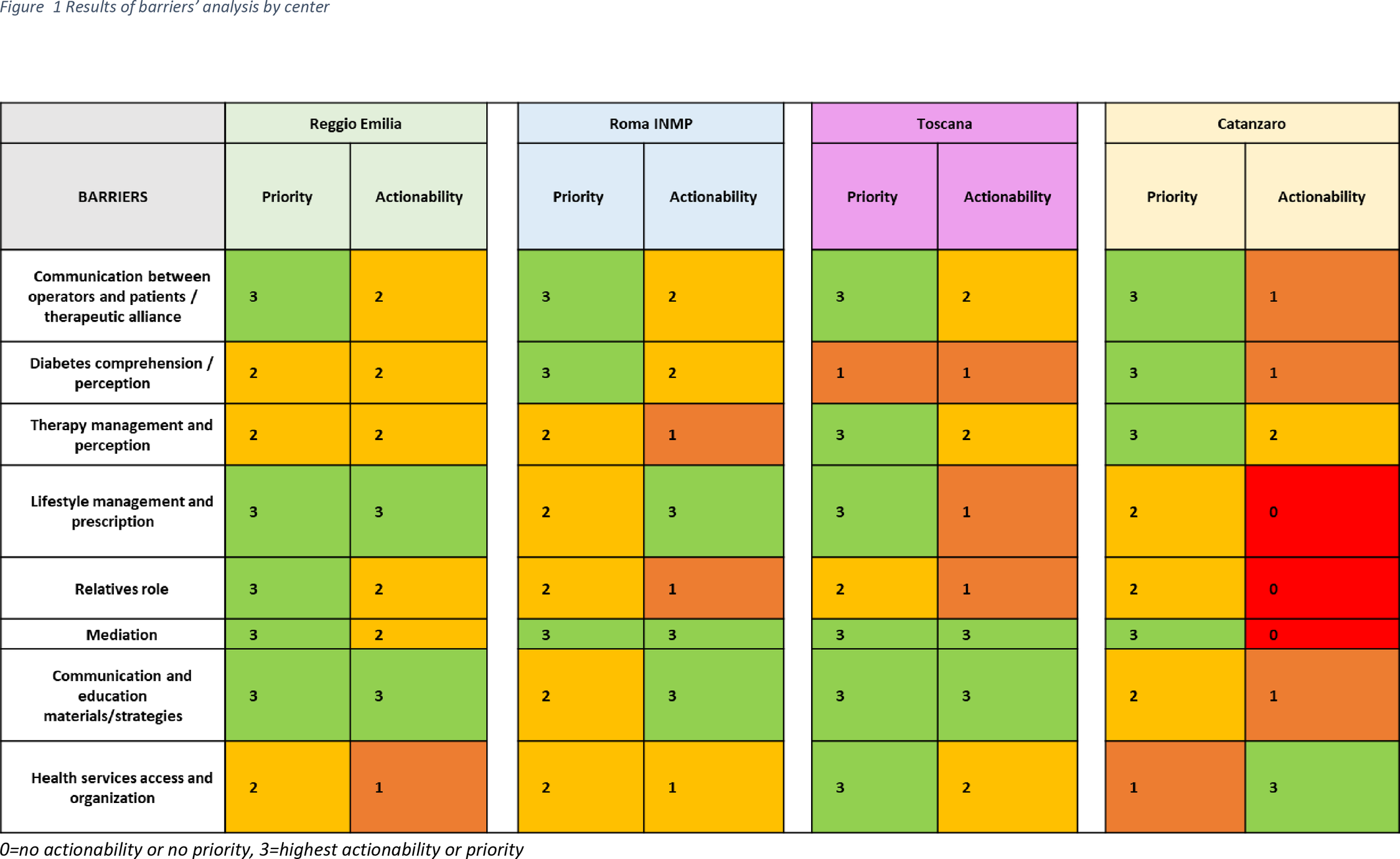

Supplement: S1 Fig — (TIF) [file pone.0317994.s002.tif]
