# Supplementary material for: A randomized trial to evaluate a complex, co-created, culture-sensitive intervention to promote healthy lifestyles and compliance to therapy in immigrants with type 2 diabetes: A protocol of a multicenter Italian study
Source: PLoS One. 2025 Feb 24;20(2):e0317994. doi: 10.1371/journal.pone.0317994 (PMC11849826; doi:10.1371/journal.pone.0317994)
Supplement: S1 File — (PDF) [file pone.0317994.s003.pdf]

PROTOCOL

EXPERIMENTAL STUDY ON NON-PHARMACOLOGICAL INTERVENTION

**Study title:**	*CarDIo-metABolic diseases in immigrants and ETHnIC minorities: from epidemiology to new prevention strategies.*
**Study ID:**	DIABETHIC
**Eudract number:**	
**Protocol version:**	V 1.1
**Date:**	02/12/2022
**Funding:**	Submitted for funding to the call PNRR: M6/C2_CALL 2022, Ministero della Salute, funded by the European Union.
**Promoting centre:**	*AZIENDA OSPEDALIERO-UNIVERSITARIA CAREGGI, Largo G.A. Brambilla 3, 50134, Firenze*
**Coordinating centre:**	*Azienda Ospedaliero-Universitaria Careggi, Dipartimento Medico-Geriatrico, SOD Medicina dello Sport, Firenze*
**Principal investigator:**	*Modesti Pietro Amedeo, SOD Medicina dello Sport*
**Other investigators from the Coordinating centre**	*Boddi Maria*<br>*Pellegrino Alessio*

List of participants

**Operative Unit**	*Istituto Nazionale salute, Migrazioni e Povertà (INMP)*
**Operative Unit**	*Istituto in tecnologie avanzate e modelli assistenziali in oncologia - AUSL Reggio Emilia*
**Operative Unit**	*Azienda Ospedaliero Universitaria Mater Domini, Catanzaro*

Contact informations

**Promoter contact name**	*Prof. Pietro Amedeo Modesti*
**Contact Name for Pharmacovigilance**	*N/A*

PROTOCOL APPROVAL

The investigators:
- approve the current protocol version;
- declare that the study will be conducted in compliance with the current version of the protocol.

Prof. Pietro Amedeo Modesti	2/12/2022<br>Date
Prof. Maria Boddi	2/12/2022<br>Date
Dott. Alessio Pellegrino	2/12/2022<br>Date

Index

Background and rationale 5
Study aims 6
  Primary aim 6
  Secondary aims 7
Study design 7
Study population (addendum for operational protocol) 8
Eligibility criteria 8
  Inclusion criteria 8
  Exclusion criteria (addendum for operational protocol) 9
Interventions 9
  Intervention A (addendum for operational protocol) 9
  Intevention B (es. comparison) 11
  Adherence to intervention (addendum for operational protocol) 11
  Benefit-risk assessment for the population (addendum for operational protocol) 11
  Participants' withdrawal (addendum for operational protocol) 12
  Premature study termination or suspension (addendum for operational protocol) 12
  Definition of study termination (addendum for operational protocol) 12
Study endpoints (addendum for operational protocol) 12
  Primary endpoint 12
  Secondary endpoint 12
Study plan 13
  Study timeline (addendum for operational protocol) 13
  Sample size 14
  Screening of potentially eligible patients (addendum for operational protocol) 14
  Recruitment process (addendum for operational protocol) 14
  Intervention assignment 15
  Blinding (addendum for operational protocol) 15
Data processing and conservation (addendum for operational protocol) 15
  Data collection 15
  Data management 15
  Data retention 16
  Protocol deviations 16
Statistical plan 16
  Subgroup analysis 17
Safety Management (addendum for operational protocol) 17
  Definitions 17
  Adverse events data collection and management, ... 17
  Abnormal laboratory findings 17
Administrative Information (addendum for operational protocol) 17

Fundings ... 17
Insurance compensation ... 17
Independent Data Monitoring Committee (IDMC) and procedure revisions ... 17
Protocol amendments ... 18
Ethics ... 18
Informed consent ... 18
Confidentiality ... 18
Conflict of interest ... 18
Accountability and dissemination policy ... 18
Role of the sponsor and investigators, data property, and publication policy ... 18
Bibliography ... 19

Background and rationale

Diabetes burden of disease

Cardiovascular disease reduced in most European countries during the last 2 decades (Nichols 2013) although large differences exist between migrants and host populations (Pacelli 2016). In Europe, type 2 diabetes and hypertension among refugee and migrant populations were found highly prevalent and often undiagnosed and uncontrolled leading to a higher risk of CVDs.

Hypertension is especially prevalent among migrants from the African region (Modesti, Reboldi et al. 2016) who often experience obstacles in accessing medical care and low levels of awareness and control (Agyemang 2018). Type 2 diabetes is highly prevalent in all migrant groups living in Europe. Compared with the host populations, the odds for type 2 diabetes is 3.7 for South Asian, 2.7 for Middle Eastern and North Africans, 2.6 for Sub-Saharan Africans, 2.3 for Western Pacific, and 1.3 for Central Americans (Meeks 2016). Studies on South Asian immigrants with diabetes in Italy (Campostrini 2019) also showed that they have more difficulties in reaching glycaemic control. It is not clear if this is due to genetic background, diet, and lifestyle, or to compliance with therapy. Sociocultural factors (cultural beliefs and traditions) affect self-management, dietary habits, medication adherence, and are often causes of scepticism about the benefits of medication (Beune 2019; Alzubaidi 2015).

Notwithstanding these findings, the recent experience of data collection for the WHO Europe Region highlighted the lack of structured data collection systems about these aspects (WHO 2022). In particular, data available in Italy mostly refer to population studies conducted at a local or regional level. (Fedeli 2018; Modesti 2021; Modesti, Calabrese et al. 2017; Modesti, Castellani et al. 2017)

Immigrant communities and health promotion

In high-income countries, lifestyle intervention trials resulted in significant improvement in diabetes incidence and glycaemic outcomes. (Hopper 2011; Schellenberg 2013). Nevertheless, interventions developed to improve diet, physical activity and compliance to therapy in immigrant hard-to-reach populations, showed variable effectiveness depending on the context and type of intervention (Modesti, Galanti et al. 2016). No trials were conducted among immigrant communities in Italy where the close interaction between social and health problems finds a crucial point in the immigrant population not registered in the Regional Health System. The current system in Italy provides that these subjects can be included in the process of care of the Regional Health System by issuing an STP code ("Straniero Temporaneamente Presente"). In this way, the subject can be assisted in relation to the diagnosed problems. However, this requires recognition and diagnosis of the condition that is usually carried out in emergency conditions (during hospitalization for myocardial infarction or stroke) due to barriers in accessing primary care services. In this context, diagnosing high-risk but long-term asymptomatic diseases such as diabetes mellitus or hypertension became more difficult, with a negative impact on primary and

secondary prevention opportunities, and consequently on the burden of diseases in these population subgroups.
The impact of context e minority-specific factors on the effectiveness of prevention strategies highlighted the importance of involving communities in defining health promotion interventions. The co-creation process includes the final users together with other stakeholders to assess health priorities and to define the best strategies to reach individual health aims, removing informal barriers and supporting cultural adaption, thus, finally, reducing inequalities. (Janamian 2016; Leask 2019)

The "Cardio-metabolic diseases in immigrants and ethnic minorities: from epidemiology to new prevention strategies." project

The availability of reliable and routinely collected data on chronic diseases in immigrant populations, the reaching of minorities not registered in the Regional Health System, and evidence-based lifestyle preventive interventions effective among different minorities may contribute to reducing the burden of chronic diseases on the National Health System.
To face this challenge, four research centres located in four different Italian Regions joined a research-intervention project founded by the Italian Ministry of Health within the Piano Nazionale di Ripresa e Resilienza (PNRR) funding program.
Within the first aim of the project, the National Institute for Health, Migration and Poverty (INMP) with the collaboration of the Italian Regions will explore the feasibility of an integrated data collection from the health systems of four regions on chronic disease indicators among immigrant population.
The second aim is focused on the strategies supporting the emergence of the unregistered populations, which remains an unexplored point in Italy as in many areas of Europe. (Woodward 2014)
Finally, the third aim concerns the co-creation of a culture-sensitive intervention to promote healthy diet, and physical activity, and to improve compliance to therapies in immigrants with type 2 diabetes and assess its effectiveness in different minorities at a multicentre level.

Study aims

Primary aim

The aim of the study is to evaluate the efficacy of the co-created, culture-sensitive intervention to promote healthy diet, physical activity, and to improve compliance to therapeutic protocols in immigrants with type 2 diabetes.

The evaluation concerns the efficacy of the intervention in the change of glycated haemoglobin 12 months after recruitment.

Secondary aims

Secondary objectives are:

- Description of changes in:
    - anthropometric measures (BMI, waist circumference)
    - dietary habits
    - physical activity habits
    - lipid profile
    - compliance with individual therapeutic protocols

Study design

The study is a multicentre randomized controlled open trial. The study design is reported in Figure 1.
Immigrants with type 2 diabetes registered at the Italian Regional Health Service or provided with STP code will be recruited.
Across the four recruiting centres, at least four different immigrant communities will be involved.
Endocrinology and Diabetology physicians at Diabetes clinics of both the National Health Service and non-governmental organizations (NGO) will do the recruitment.
Eligible immigrants who accept to participate will sign the consent during routine visits at Diabetes clinics and will be randomized to the control or intervention group.

The intervention group will receive a co-created culture-tailored intervention of health promotion aimed to increase healthy diet, physical activity and compliance with treatment protocols for diabetes.

The control group will receive the health promotion activities routinely performed by Diabetes clinics at the recruiting centres.

The enrolment phase will last 4 months.
The intervention will last at least 3 months.
Follow-up visits will be performed 12 months after recruitment.
The study period will be between 01/01/2023 and 31/12/2025, compatibly with the trend of the COVID-19 emergency.

Questionnaires about diet and physical activity will be collected at baseline and 12-month visits, as well as anthropometric measures.
Blood and urine samples will be collected at baseline and at 12 months to measure glycated haemoglobin and other biomarkers.

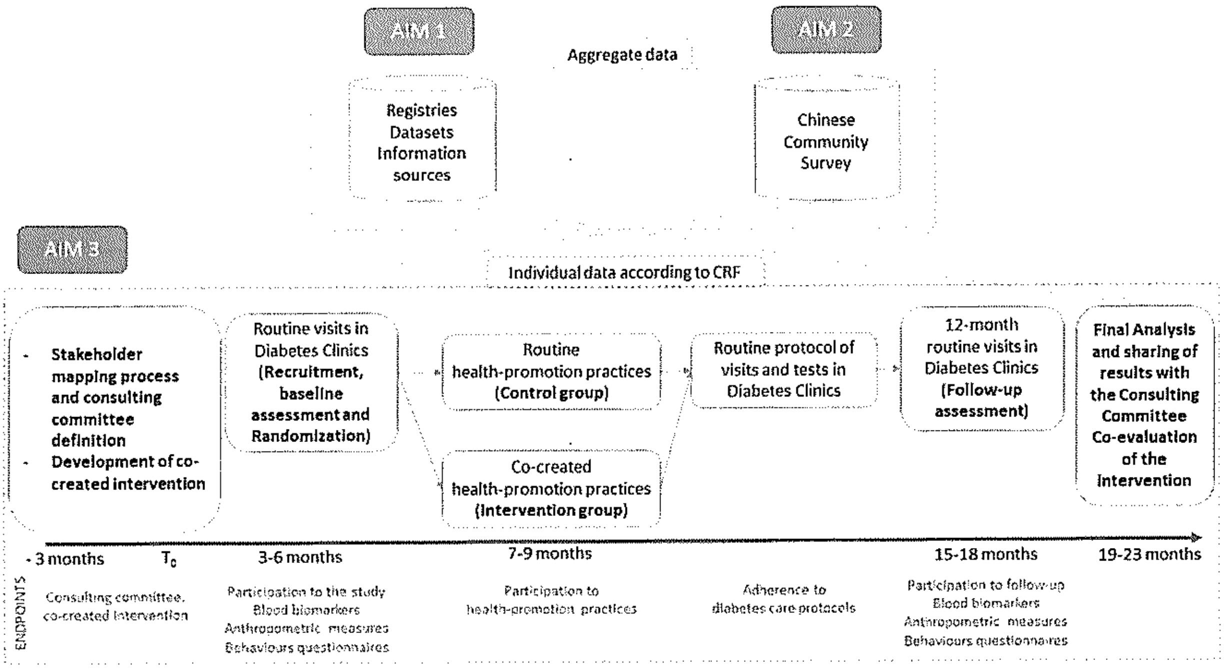

Figure 1. DiabEthic study design.

Study population (addendum for operational protocol)

The study will be performed within outpatients and inpatients' Diabetes clinics of the four Italian recruiting centres: Azienda Ospedaliero-Universitaria Careggi (Florence), Istituto Nazionale salute, Migrazioni e Povertà (INMP, Rome); Istituto in tecnologie avanzate e modelli assistenziali in oncologia-AUSL Reggio Emilia; Azienda Ospedaliero Universitaria Mater Domini (Catanzaro).

In each centre immigrants attending routine visits will be assessed for eligibility and, when appropriate, informed of the study and asked to participate. During the enrollment phase, cultural mediators will be involved if needed (subjects who do not speak or understand the Italian language will not be excluded). Due to the different ethnic compositions of the populations living in each centre, different minorities will be involved.
Intervention, control and follow-up will be performed within each centre.
According to the power analysis, at least 200 patients should be recruited overall.

Eligibility criteria

Inclusion criteria

Eligibility criteria include:

- Immigrant status: Self-identification of being born in High migration pressure countries from parents born in High migration pressure countries (Carletti 2009);
- Age ≥ 18 years
- Type 2 diabetes newly diagnosed or with HbsA1c > 8% in the last assessment within 24 months before the visit

Exclusion criteria (addendum for operational protocol)

Exclusion criteria include:

- Patients who will not provide the informed consent
- Patients aged under 18.
- Patients with HbsA1c ≤ 8% in the last assessment within 24 months before the visit.
- Severe psychiatric disorders
- Pregnant women
- Critical illness
- Impaired cognitive or physical ability that could make the intervention not feasible, as judged by clinical staff members.

Interventions

Intervention A (addendum for operational protocol)

The intervention will include key elements of proven efficacy for health promotion combined and adapted to each community through a co-design and co-creation process, involving service providers and end-users.

Stakeholders will include professionals involved in diabetes care and providing services to hard-to-reach populations, immigrants with diabetes (the final user), relevant people of the communities and professionals of the third sector. A combination of different methodologies (focus group, consultations, stakeholder meetings, and qualitative interviews among the others) will be used by each centre to co-create a community-tailored intervention [Leask CF 2019] adapted to the local context of each centre with a different implementation of key elements:

a) Dietary habit: the key elements will be theoretical dissemination of information on healthy diet (i.e. cookbooks with healthy and easy-to-make recipes), and courses on healthy cooking targeted to family and factory cooks (i.e. how to cook healthy dishes respecting the preferences and the taste of the community of origin, but feasible with ingredients available in the hosting region).
b) Physical activity: the key elements will be the promotion of organized physical activity compatible with the daily time restrictions of the patients, promotion of non-organized physical activity (i.e. analysing the possibilities of moving home-work on foot or by bicycle and using an opportunity map that shows parks and possibilities for spontaneous physical

activity), theoretical dissemination of information on adapted physical activity (i.e. lectures with sports instructors or healthcare professional with specific expertise). Use of apps to measure physical activity will be considered.

c) Adherence to treatments: tools favouring compliance and glucose measuring, as well as culture-tailored information material on diabetes and its health implications will be used.

The intervention will last a minimum of 3 months and will include at least one individual counselling (IC), one family session, and at least two group sessions (FS) for dietary and physical activity courses. Mandatory elements for the intervention and a non-exhaustive list of optional elements, as well as, elements that can be modulated in the co-creation phase are reported by topic in Table 1.

Table 1: Mandatory and optional elements of the intervention and elements that can be modulated in the co-creation phase.

**Topic**	**Mandatory elements**	**Optional elements**	**Co-creation modular elements**
**Diet**	- culturally tailored cookbooks with healthy and easy-to-make recipes		- contents of cookbooks
	- personalised dietary plan		- food, recipes to be included<br>- daily timeline of eating opportunities
		- cooking courses for formal and informal cooks including culturally tailored healthy dishes feasible with ingredients available in the hosting region	- Recipes' to be included<br>- Provider of the cooking courses<br>- Target of the cooking courses
**Physical activity**	- theoretical dissemination of information on adapted physical activity		- type of sports or physical activity to be included
	- opportunity map including cycle lines, parks, public physical activities facilities with free access		- Facilities and other elements of interest to be included in the map<br>- information of interest on each facility
		- promotion of non-	- solutions based on

		organized physical activity using an opportunity map	specific needs and barriers to be considered
		- organization of courses on structured physical activity	- methodology of courses delivery and sports to be included
**Adherence to treatments**	- Cultural mediation (2 hours per patients		- Mediators to be involved<br>- Setting for mediation
		- courses to health professional or informal caregivers	- content of the courses<br>- caregivers to be involved

Intevention B (es. comparison)

The control group will follow the usual care practice of each Diabetes clinic including, at least, multi-language information material, pre-planned routine visits as recommended by National guidelines (SID, AMD 2021), and a cultural mediation intervention when required by the diabetologist.

Adherence to intervention (addendum for operational protocol)

The adherence to intervention and control will be monitored through clinical records and attendance registers fulfilled by the physician responsible for each phase of the intervention.

Benefit-risk assessment for the population (addendum for operational protocol)

Since the intervention and control group will undergo routinely proposed clinical and preventive interventions (i.e., routine visits and tests), no additional risks for the study participants are foreseen.
Benefits from the project and study results are foreseen at different levels, as described in the proposal.
The knowledge about how risk factors are acting in our Country on major chronic diseases in immigrants as well as the evidence about methods to contact hard-to-reach populations to propose preventive intervention and diabetes care can directly be used in planning tailored interventions for immigrant communities at particularly high risk of chronic diseases.
The results of the trial will pave the way to co-create interventions applicable to local communities of immigrants in any Italian context
The three levels of knowledge acquired with this project will be immediately transferable to three levels. First, the information on the current levels of integration of knowledge in the treatment process is relevant for planning purposes (implications for public health).

Second, the experience gained by the staff who will conduct the study will allow a direct transfer of knowledge in the local realities where the study is conducted with an important advancement in clinical practice (implications for clinical practice).
Third, the results of the clinical trial on culturally mediated interventions shared with migrant communities will allow a transfer of knowledge even at the level of elusive ethnic communities (implications for patient care).

Participants' withdrawal (addendum for operational protocol)

All the participants may withdraw from the study at any moment, and participation and compliance with follow-up will be monitored. Access to routine management will be guaranteed to each individual.

Premature study termination or suspension (addendum for operational protocol)

There are no anticipated stopping rules.

Definition of study termination (addendum for operational protocol)

For each participant, the study stops after participation in the 12-month routine visit.

Study endpoints (addendum for operational protocol)

Primary endpoint

The primary endpoint is the change in glycated haemoglobin (HbA1c; mmol/mol) between the recruitment and the 12-month follow-up visit assessed through the collection of fasting blood and urine samples.

The difference between groups of the mean change (and standard deviation) in glycated haemoglobin will be used to assess the effectiveness of the intervention.

Secondary endpoint

Secondary endpoints include:

- Biomarkers related to lipid profile assessed through the collection of fasting blood samples: triglycerides (mmol/l), LDL (mmol/l), HDL (mmol/), total Cholesterol (mmol/l), free fatty acids (mmol/l), anthropometric measures (height, weight, BMI, waist circumference) and health related behaviours.
- Anthropometric measures: weight, height and waist circumference, bioelectrical impedance analysis (BIA).
- Systolic and diastolic blood pressure.
- Physical activity and dietary habits self-reported data.
- increase in adherence to Mediterranean Diet

- restriction of daily alcohol consumption (<160 kcal);
- compliance with WHO recommended exercise goals
- Indicators of compliance with individual therapeutic protocols in terms of number of attended visits, number of tests of renal function and glycaemic control, and drug prescription.

Blood and urine samples and anthropometric measures will be collected at baseline and 12-month follow up visits.
Physical activity and dietary habits data will be collected through a questionnaire on lifestyle, dietary habit and physical activity fulfilled by each participant during routine visits in Diabetes clinics. The questionnaires used to assess dietary habits will be the Mediterranean Diet Score, (MedDietScore).(Panagiotakos 2007) The questionnaire used to assess physical activity will be the International Physical Activity Questionnaire - Short Form, IPAQ-SF. (Mannocci 2010)
Therapeutic adherence will be assessed at baseline and follow up visits using the Diabetes Mellitus Treatment Adherence Scale (DMTAS). (Hou 2021)
Descriptive analysis of mean (or median) changes with relative SD (or IQR) and the proportion of patients reaching therapeutic/recommended targets will be used.

Study plan

Study timeline (addendum for operational protocol)

The enrolment phase will last 4 months.
The intervention will last at least 3 months.
Questionnaires about diet and physical activity will be collected at baseline and 12-month visits, as well as anthropometric measures. The study timeline is reported in Figure 2.
Blood and urine samples will be collected at baseline and at 12 months to measure glycated haemoglobin and other biomarkers

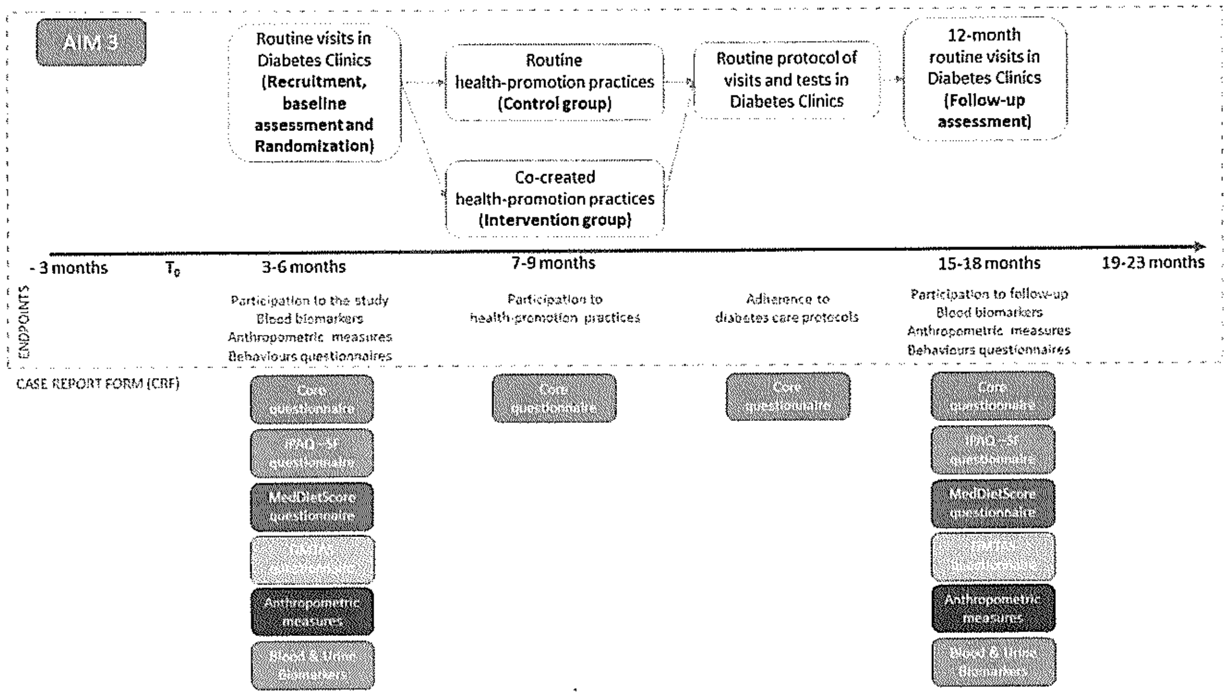

Figure 2: Study timeline and data collection

Sample size

To explore the efficacy of a culturally adapted diabetes education model in improving health literacy and self-care (primary endpoint) in immigrant patients with type 2 diabetes. The intervention is expected to provide a 0.5% higher reduction in the concentration of HbA1c compared to the usual care at a 12-month follow-up, with a positive effect on the risk of CVD events (Selvin 2004; Mitsios 2018). Through a change in health-related behaviours, it is also expected to reduce overweight and increase vegetable intake and physical activity, with a positive impact on future risk of non-communicable diseases and quality of life (GBD 2019 Risk Factors Collaborators 2020). To have a power of 80%, considering an alpha of 0.05 (Islam 2013), in order to detect a minimum significant reduction of 0.5% in the glycated haemoglobin in the intervention group compared to the control group at least 200 participants are needed (considering a SD in both group of 1.3) (Bellary 2008).

Screening of potentially eligible patients (addendum for operational protocol)

Physicians working at Diabetes clinics during routine visits will perform the eligibility assessment. Eligible patients will be informed on the study and asked to participate, collecting the informed consent.

Recruitment process (addendum for operational protocol)

The recruitment will occur within Diabetes clinics routine visits in each centre.

Consecutive enrolment of potentially eligible patients will be performed over a four-month period. Whether low participation rates will occurs, a preliminary eligibility assessment on medical records of Diabetes clinics will be performed, and potentially eligible patient will be actively contacted. Pre-recruitment eligibility criteria assessment from third sector organizations caring for undocumented migrants may also support active recruitment.

Intervention assignment

Block randomization (1:1 ratio) will be computerized and conducted in each centre to balance the ethnic composition of the intervention and control groups. Members of the same household, whether identified as cohabitant during the recruitment visit, will be assigned at the same arm.

Blinding (addendum for operational protocol)

The randomization arm will not be masked to the participant nor to the investigator. The person who enrols will be blinded to the random sequence until each patient will sign the informed consent (allocation concealment).

Data processing and conservation (addendum for operational protocol)

Data collection

The clinical information will be locally collected using the GDPR Compliant electronic platforms adopted by the recruiting centres for collecting clinical data allowing randomization.
Periodic monthly monitoring of recruitment, randomization procedures and follow-up will be collected on aggregated data reporting: number of patient contacted, refusal to participate (by sex and community), randomization (by gender, community and age group <50 and >50), presence at follow up visit.

Biomarkers will be assessed through analysis of blood and urine samples collected following standard procedures of the Diabetes clinics and analysed for glycaemia, by the authorised clinical laboratories of each recruiting centre.

Questionnaires used to assess dietary habits and physical activity will be the Mediterranean Diet Score, (MedDietScore) (Panagiotakos 2007) and the International Physical Activity Questionnaire - Short Form, IPAQ-SF. (Mannocci 2010)
Anthropometric measures will be collected following standard procedures during routine visits in Diabetes clinics with validated weight and height scales and bioelectrical impedance analysis (BIA).
Therapeutic adherence will be assessed at baseline and follow up visits using the Diabetes Mellitus Treatment Adherence Scale (DMTAS). (Hou 2021)

Data management

Data will be entered with manual data entry on GDPR-compliant digital platforms as in use in the recruiting centres. The personal information will be stored locally and will be subject to technical

and organizational safeguards. For each participating subject, the system will generate a pseudo-anonymization code with the specification of the recruitment centre, as no individual can be identified without reference to personal information. This code will be present in each record relating to the same participant in the various tables of the CRF. Each centre will carry out quality controls of the data entered through crosschecks with the information present in the original sources (paper questionnaires, medical records).
Pseudo-anonymised datasets will be sent by each recruiting centre to the centre which will perform statistical analysis (Azienda USL-IRCCS di Reggio Emilia) according to the procedures reported in the Data Transfer Agreement at the end of the recruitment end follow-up.
The final analysis will be carried out when the 12-month follow-up will be available for all participants.

Data retention

Each local Principal Investigator will be responsible for data management in participating centre while the study Principal Investigator will be responsible for data management at a central level. In the recruiting centres, the data will be stored on separate files with the relative linkage key in reserved areas protected by passwords as per company procedures; the data will be kept for 20 years. The pseudo-anonymized data will be kept for 20 years in the coordinating centre.
The centres will apply the procedures for conducting clinical studies envisaged by local IT services and evaluated by local Data Protection Officers.

Protocol deviations

Substantial changes to the protocol will be submitted for evaluation by the ethics committee of the coordinating centre and then transmitted to the local ethics committees.

Statistical plan

Descriptive statistics will be calculated for baseline characteristics. The analysis will be intention-to-treat. Before/after variations of the glycated haemoglobin, lipid profile and renal function biomarkers, and anthropometric measure will be computed and standardized if opportune. Paired and unpaired tests will be used to assess the effects of the intervention and to analyse before/after within-group and between-group differences and changes in glycated haemoglobin. Changes in lifestyle habits (both related to diet and physical activities) will be described in terms of positive or negative changes.
Linear regression models will be used to analyse the variation of anthropometric measures and assessed biomarkers.
Multilevel linear models will be performed taking into account the influence of the centre on intervention efficacy.
We will perform a mediation analysis to understand which part of the changes in outcomes (glycated haemoglobin, lipid profile, and anthropometric measures) is attributable to changes in

physical activity, changes in diet, adherence to therapies, and other direct or unmeasured effects of the intervention.
The statistician will not be blinded to group assignments.
The statistical significance level will be set at 5%, and all analyses will be performed by using Stata 16 or SPSS 28.

Subgroup analysis

Subgroup analyses will be performed according to weight status, gender, ethnicity, and socioeconomic level.

Safety Management (addendum for operational protocol)

Definitions

The intervention and control group underwent routinely proposed clinical and preventive interventions (i.e. routine visits and tests). Thus, predictable adverse events are those foreseen for routine clinical practice.

Adverse events data collection and management,

Predictable and unpredictable adverse events or potentially relevant undesirable effects will be reported and managed according to standard institutional Safety Management procedures.

Abnormal laboratory findings

Abnormal laboratory findings will be managed according to local clinical protocols.

Administrative Information (addendum for operational protocol)

Fundings

The study has been submitted for funding to the call PNRR: M6/C2_CALL 2022, Ministero della Salute, funded by the European Union. The role of the funding source is reported in the call of the Ministry of Health (Bando Piano Nazionale di Ripresa e Resilienza) available at https://ricerca.cbim.it/Documentazione.

Insurance compensation

Since the intervention and control group underwent routinely proposed clinical and preventive interventions (i.e. routine visits and tests), patients will be covered by institutional insurance of each centre. No study-specific insurance is needed.

Independent Data Monitoring Committee (IDMC) and procedure revisions

The recruitment, intervention, and follow-up phases are planned to occur within 12 to 18 months and no stopping rules are foreseen. Thus, no independent Monitoring committee will be created and no routine revisions of processes and documents are planned. Data and processes monitoring

will be performed according to requirements reported in the call of the Ministry of Health (Bando Piano Nazionale di Ripresa e Resilienza) available at https://ricerca.cbim.it/Documentazione.

Protocol amendments

Substantial changes to the protocol will be submitted for evaluation by the ethics committee of the coordinating centre and then transmitted to the local ethics committees.

Ethics

The trial must be conducted in accordance with:

- The principles of ethics as stated in the last version of the Declaration of Helsinki,
- The relevant provisions of Good Clinical Practices defined by the International Conference on Harmonisation (ICH–E6 R2, December 2016),

All ethical requirements are also reported in the call of the Ministry of Health (Bando Piano Nazionale di Ripresa e Resilienza) available at https://ricerca.cbim.it/Documentazione.

Informed consent

The eligible patients will be informed on the study and those interested in participating will be asked to sign informed consent during routine visits at Diabetes clinics. Patients aged <18 years or unable to provide a personal informed consent are not eligible for the study.

Confidentiality

The requirements for Data Protection are reported in the call of the Ministry of Health (Bando Piano Nazionale di Ripresa e Resilienza) available at https://ricerca.cbim.it/Documentazione .
A Data Protection Declaration compliant with GDPR is attached to the current protocol.

Conflict of interest

No conflict of interests to declare.

Accountability and dissemination policy

Role of the sponsor and investigators, data property, and publication policy

The Role of Sponsor and Investigators, Data Ownership Rules and Publication Policies will be compliant with FAIR Data principles and other requirements reported in the call of the Ministry of Health (Bando Piano Nazionale di Ripresa e Resilienza) available at https://ricerca.cbim.it/Documentazione.
A more detailed description will be provided in the agreement between the Ministry of Health and the coordinating centre that will be signed after the proposal approval.

Bibliography

Agyemang C, Nyaaba G, Beune E, et al. Variations in hypertension awareness, treatment, and control among Ghanaian migrants living in Amsterdam, Berlin, London, and nonmigrant Ghanaians living in rural and urban Ghana - the RODAM study. J Hypertens. 2018;36(1):169-177. doi:10.1097/HJH.0000000000001520

Alzubaidi H, Mc Narmara K, Kilmartin GM, Kilmartin JF, Marriott J. The relationships between illness and treatment perceptions with adherence to diabetes self-care: A comparison between Arabic-speaking migrants and Caucasian English-speaking patients. Diabetes Res Clin Pract. 2015;110(2):208-217. doi:10.1016/j.diabres.2015.08.006

Bellary S, O'Hare JP, Raymond NT, et al. Enhanced diabetes care to patients of south Asian ethnic origin (the United Kingdom Asian Diabetes Study): a cluster randomised controlled trial. Lancet. 2008;371(9626):1769-1776. doi:10.1016/S0140-6736(08)60764-3

Beune E, Nieuwkerk P, Stronks K, et al. Medication non-adherence and blood pressure control among hypertensive migrant and non-migrant populations of sub-Saharan African origin: the RODAM study. J Hum Hypertens. 2019;33(2):131-148. doi:10.1038/s41371-018-0120-8

Campostrini S, Carrozzi G, Severoni S, et al. Migrant health in Italy: a better health status difficult to maintain-country of origin and assimilation effects studied from the Italian risk factor surveillance data. Popul Health Metr. 2019;17(1):14. Published 2019 Nov 1. doi:10.1186/s12963-019-0194-8

Carletti P, et al. La salute della popolazione immigrata: metodologia di analisi. Progetto "Promozione della salute della popolazione immigrata in Italia. Accordo Ministero della salute/CCM - Regione Marche e coordinato dalla Regione Marche 2007–2009. Pag 122-127
Rome, Italy. 2009. Available at https://www.ccm-network.it/documenti_Ccm/prg_area5/Prg_5_Immigrati_metodologia.pdf.pdf

Fedeli U, Avossa F, Ferroni E, Schievano E, Bilato C, Modesti PA et al. Diverging patterns of cardiovascular diseases across immigrant groups in northern Italy. Int J Cardiol. 2018;254:362–7.;

GBD 2019 Risk Factors Collaborators. Global burden of 87 risk factors in 204 countries and territories, 1990-2019: a systematic analysis for the Global Burden of Disease Study 2019. Lancet. 2020;396(10258):1223-1249. doi:10.1016/S0140-6736(20)30752-2

Hopper I, Billah B, Skiba M, Krum H. Prevention of diabetes and reduction in major cardiovascular events in studies of subjects with prediabetes: meta-analysis of randomised con- trolled clinical trials. Eur J Cardiovasc Prev Rehabil 2011;18(6):813–823

Hou G, Fang Z, Cao W, et al. Development and validation of a diabetes mellitus treatment adherence scale. Diabetes Res Clin Pract. 2021;172:108629. doi:10.1016/j.diabres.2020.108629

Islam NS, Wyatt LC, Patel SD, et al. Evaluation of a community health worker pilot intervention to improve diabetes management in Bangladeshi immigrants with type 2 diabetes in New York City. Diabetes Educ. 2013;39(4):478-493. doi:10.1177/0145721713491438

Janamian T, Crossland L, Jackson CL. Embracing value co-creation in primary care services research: a framework for success. Med J Aust. 2016;204(7 Suppl):S5-S11. doi:10.5694/mja16.00112

Leask CF, Sandlund M, Skelton DA, et al. Framework, principles and recommendations for utilising participatory methodologies in the co-creation and evaluation of public health interventions. Res Involv Engagem. 2019;5:2. Published 2019 Jan 9. doi:10.1186/s40900-018-0136-9

Mannocci A, Masala D, Mei D, Tribuzio AM, Villari P, LA Torre G. International Physical Activity Questionnaire for Adolescents (IPAQ A): reliability of an Italian version. Minerva Pediatr (Torino). 2021;73(5):383-390. doi:10.23736/S2724-5276.16.04727-7

Meeks KA, Freitas-Da-Silva D, Adeyemo A, et al. Disparities in type 2 diabetes prevalence among ethnic minority groups resident in Europe: a systematic review and meta-analysis. Intern Emerg Med. 2016;11(3):327-340. doi:10.1007/s11739-015-1302-9

Modesti PA, Calabrese M, Malandrino D, Colella A, Galanti G, Zhao D. New findings on type 2 diabetes in first-generation Chinese migrants settled in Italy: Chinese in Prato (CHIP) cross-sectional survey. Diabetes Metab Res Rev. 2017;33(2).

Modesti PA, Castellani S, Calabrese M, Malandrino D, Zhao D. Comparison of type 2 diabetes prevalence in Chinese migrants vs Caucasians and new perspectives for screening of cerebrovascular disease in Chinese: A proof of concept study. Diabetes Res Clin Pract. 2017;130:196-203.

Modesti PA, Galanti G, Cala' P, Calabrese M. Lifestyle interventions in preventing new type 2 diabetes in Asian populations. Intern Emerg Med. 2016;11(3):375-84.

Modesti PA, Marzotti I, Calabrese M, Stefani L, Toncelli L, Modesti A, Galanti G, Boddi M. Gender differences in acculturation and cardiovascular disease risk-factor changes among Chinese immigrants in Italy: Evidence from a large population-based cohort. Int J Cardiol Cardiovasc Risk Prev. 2021;11:200112

Modesti PA, Reboldi G, Cappuccio FP, et al. Panethnic Differences in Blood Pressure in Europe: A Systematic Review and Meta-Analysis. PLoS One. 2016;11(1):e0147601. Published 2016 Jan 25. doi:10.1371/journal.pone.0147601

Nichols M, Townsend N, Scarborough P, Rayner M. Trends in age-specific coronary heart disease mortality in the European Union over three decades: 1980-2009. Eur Heart J. 2013;34(39):3017-3027. doi:10.1093/eurheartj/eht159

Pacelli B, Zengarini N, Broccoli S, et al. Differences in mortality by immigrant status in Italy. Results of the Italian Network of Longitudinal Metropolitan Studies. Eur J Epidemiol. 2016;31(7):691-701. doi:10.1007/s10654-016-0177-z

Panagiotakos DB, Pitsavos C, Arvaniti F, Stefanadis C. Adherence to the Mediterranean food pattern predicts the prevalence of hypertension, hypercholesterolemia, diabetes and obesity, among healthy adults; the accuracy of the MedDietScore. Prev Med. 2007;44(4):335-340. doi:10.1016/j.ypmed.2006.12.009

Schellenberg ES, Dryden DM, Vandermeer B, Ha C, Korownyk C. Lifestyle interventions for patients with and at risk for type 2 diabetes: a systematic review and meta-analysis. Ann Intern Med 2013;159(8):543–551

Selvin E, Marinopoulos S, Berkenblit G, et al. Meta-analysis: glycosylated hemoglobin and cardiovascular disease in diabetes mellitus. Ann Intern Med. 2004;141(6):421-431. doi:10.7326/0003-4819-141-6-200409210-00007

SID, AMD Linea Guida della Società Italiana di Diabetologia (SID) e dell'Associazione dei Medici Diabetologi (AMD) La terapia del diabete mellito di tipo 2 pubblicata nel Sistema Nazionale Linee Guida Roma, 26 luglio 2021 Available at https://snlg.iss.it/wp-content/uploads/2021/07/LG_379_diabete_2.pdf

Woodward A, Howard N, Wolffers I. Health and access to care for undocumented migrants living in the European Union: a scoping review. Health Policy Plan. 2014;29(7):818-830. doi:10.1093/heapol/czt061

World report on the health of refugees and migrants. Geneva: World Health Organization; 2022. Licence: CC BY-NC-SA 3.0 IGO
